# The need for an active role of the clinical radiologist in the management of the HIV-infected patients

**DOI:** 10.4102/sajr.v21i2.1226

**Published:** 2017-11-14

**Authors:** Colin N. Menezes

**Affiliations:** 1 Division of Infectious Diseases, Department of Internal Medicine, Chris Hani Baragwanath Academic Hospital, South Africa; 2 Department of Internal Medicine, School of Clinical Medicine, Faculty of Health Sciences, University of the Witwatersrand, South Africa

South Africa has the highest human immunodeficiency virus (HIV) prevalence rates worldwide. HIV infection rates have risen from 10.3% of the total population in 2002 to rates as high as 12.7% in 2016. The total number of individuals living with HIV infection increased from an estimated 4.72 million in 2002 to 7.03 million in 2016.^1^ Over time, HIV infection has become a chronic manageable disease with improved access to antiretroviral therapy in both public and private sectors, allowing individuals to live longer and lead healthy lives. Acquired immunodeficiency syndrome (AIDS)-related deaths have dropped from 39.6% of the total population in 2006 to 27.9% in 2016.^1^

Understanding the spectrum of complex and protean manifestations of HIV and AIDS-related diseases are crucial for a clinical radiologist because, despite specific cell tropism, patients may present with pathology in nearly every organ. The CD4+ mononuclear cells and activated CD4+ T lymphocytes are the main targets for this virus. When these cells become depleted, as a result of viral replication and cell death, patients develop AIDS.^2^ HIV-infected patients may present with opportunistic infections and malignancies, albeit occasionally, in an atypical or unusual manner, depending on the CD4+ T-lymphocyte count. As a consequence, making a diagnosis is often challenging and clinicians may rely on the assistance of imaging. This may result in a delay in diagnosis and patients receiving inappropriate treatment. The assistance of a clinical radiologist in the management of HIV-infected patients is of great importance. For example, a plain chest X-ray is an easy, first investigation to perform and interpret in an HIV-infected patient presenting with unexplained respiratory or constitutional symptoms. However, when a chest X-ray is normal, a chest CT scan is much better in identifying and characterising conditions such as early interstitial lung disease, lymphadenopathy and pulmonary nodules. This, however, requires the aid of a radiologist.^3^

A patient may present with neurological symptoms together with CT brain findings that reveal focal brain lesions. These are often difficult to interpret radiologically. In developed countries, toxoplasma encephalitis is the most common cause of focal brain lesions, followed by primary central nervous system lymphoma. In this country, tuberculosis is the most common presumed diagnosis. The microbiological diagnosis of tuberculosis from cerebrospinal fluid is often difficult. Cerebral toxoplasmosis is also a common opportunistic infection and the ability to diagnose it is important because it responds well to treatment. The role of the clinical radiologist is to detect and characterise focal lesions and aid the clinician in managing their patients. CT and MRI scans may be non-specific. There may be difficulty in differentiating tuberculomas, lymphomas or cerebral abscesses. An experienced radiologist who understands the clinical history can pick up subtle rim-enhancing abscess, particularly in the basal ganglia.^4,5,6^

Radiologists depend on their ability to recognise enhancement of such lesions which usually signifies the presence of inflammation. Clinicians, on the contrary, tend to use steroid therapy whilst awaiting the imaging results. Clinicians need to be aware that steroids blunt the inflammatory response and may convert enhancing lesions to non-enhancing lesions, affecting the radiological diagnosis.^6^ With the introduction of antiretroviral therapy, an immune reconstitution inflammatory syndrome (IRIS) is another important complication that radiologists need to be cognisant of in the management of HIV-infected patients. Some patients may develop a paradoxical worsening of their symptoms or an unmasking of symptoms after the initiation of treatment. IRIS is a diagnosis of exclusion. A rise in CD4 lymphocyte count and a decrease in viral load are common findings associated with IRIS.

Therefore, radiological findings need to be understood in the context of the clinical and laboratory findings, emphasising the holistic role of a radiologist in the management of HIV-infected patients.^7^ There is a growing need for medical practice to become increasingly interdisciplinary. In addition to working with clinicians, radiologists need to understand the clinical features, natural history and treatments of HIV infections that they are required to investigate. A high level of technical training alone is not sufficient enough to adequately contribute to the diagnosis and management of conditions in patients with HIV infection.
